# Reef fishes weaken dietary preferences after coral mortality, altering resource overlap

**DOI:** 10.1111/1365-2656.13796

**Published:** 2022-08-16

**Authors:** Robert F. Semmler, Nathan J. Sanders, Paul J. CaraDonna, Andrew H. Baird, Xin Jing, James P. W. Robinson, Nicholas A. J. Graham, Sally A. Keith

**Affiliations:** ^1^ Lancaster Environment Centre Lancaster University Lancaster UK; ^2^ University of Texas Marine Science Institute Port Aransas Texas USA; ^3^ Department of Ecology and Evolutionary Biology University of Michigan Ann Arbor Michigan USA; ^4^ Chicago Botanic Garden Glencoe Illinois USA; ^5^ Australian Research Council Centre of Excellence for Coral Reef Studies James Cook University Townsville Queensland Australia; ^6^ State Key Laboratory of Grassland Agro‐Ecosystems and College of Pastoral Agriculture Science and Technology Lanzhou University Lanzhou China

**Keywords:** bottom‐up effects, coral bleaching, dietary preferences, foraging behaviour, resource partitioning

## Abstract

1. The direct and indirect effects of climate change can affect, and are mediated by, changes in animal behaviour. However, we often lack sufficient empirical data to assess how large‐scale disturbances affect the behaviour of individuals, which scales up to influence communities.
2. Here, we investigate these patterns by focusing on the foraging behaviour of butterflyfishes, prominent coral‐feeding fishes on coral reefs, before and after a mass coral bleaching event in Iriomote, Japan.
3. In response to 65% coral mortality, coral‐feeding fishes broadened their diets, showing a significant weakening of dietary preferences across species.
4. Multiple species reduced their consumption of bleaching‐sensitive *Acropora* corals, while expanding their diets to consume a variety of other coral genera. This resulted in decreased dietary overlap among butterflyfishes.
5. Behavioural changes in response to bleaching may increase resilience of coral reef fishes in the short term. However, coral mortality has reduced populations of coral‐feeders world‐wide, indicating the changes in feeding behaviour we document here may not be sufficient to ensure long‐term resilience of butterflyfishes on coral reefs.

## Graphical abstract

This work highlights important bottom‐up effects of coral bleaching on the specificity of reef fish diets. This simultaneous dietary expansion across species follows expectations from optimal foraging theory and is driven by a weakening of dietary preferences in response to food loss. These changes can significantly alter resource overlap. Photo of a feeding *Chaetodon trifascialis* individual (credit: Sally Keith).

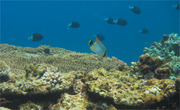

## 1 INTRODUCTION

Disturbances, including those that result from climate change, can alter the behaviour of individuals, reshape the dynamics of populations and alter the structure of communities (Sih et al., 2011, Van Buskirk, 2012). One important pathway for behavioural change is through changes in food availability (‘bottom‐up effects’, Wilson et al., 2013). For example, assuming constant competitor abundance, optimal diet or optimal foraging models predict that when food availability is low, foragers should expand their dietary breadth (Emlen, 1966). Under such conditions, preference for particular prey or resources should weaken, as consumers cannot afford to ignore lower‐quality foods when food resources become scarce (Owen‐Smith, 1994; Rödel et al., 2004). However, an increase in dietary breath may not necessarily stem from weakening dietary preferences and could instead simply reflect changes in relative prey or resource abundance. For any population, determining whether prey preferences decline after disturbance requires direct estimation of the strength of dietary preferences as food availability declines.

One important consideration of dietary expansion (i.e. a decline in dietary preferences) is its potential to affect resource overlap. The simultaneous expansion of consumer diets in response to reduced food availability could lead to increased resource overlap, as niche breadth and overlap are strongly correlated (Del Moral, 1985; Økland, 1990; Watson, 1980). Although expanded diets may benefit some species, increased resource overlap could lead to competitive exclusion, as greater partitioning permits more stable coexistence (Chesson, 2000). Quantifying the link between disturbance‐induced dietary expansion and subsequent resource overlap could help predict how populations and communities might respond to disturbances, such as thermal bleaching and mortality of tropical coral reefs.

An increasingly prevalent severe climatic disturbance is thermal bleaching and subsequent mortality of tropical coral reefs (Glynn, 1993). Coral bleaching events are five times as frequent as they were in the early 1980s (Hughes et al., 2018) and can cause substantial mortality in a diverse, yet sensitive ecosystem over extensive spatial scales (Hughes et al., 2017). Coral bleaching events often lead to biotic and functional homogenization of fish communities (Richardson et al., 2018). Bleaching also affects the behaviour of reef‐associated fishes (Gunn et al., 2022; Keith et al., 2018; Semmler, 2021). For example, the alteration of visual or olfactory cues following bleaching affects the behaviour of juvenile fishes at settlement and when they seek shelter from predators (Boström‐Einarsson et al., 2018; Coppock et al., 2015). However, while bleaching affects fish behaviour, less is known about how behavioural changes might affect resource use and overlap of fishes after disturbance events.

Bleaching‐induced coral mortality provides an (albeit unfortunate) opportunity to evaluate bottom‐up effects of disturbance on resource preference and niche overlap. Bleaching‐induced coral mortality reduces food availability for coral‐feeding fishes like butterflyfishes (Genus: *Chaetodon*; Keith et al., 2018). For many butterflyfish species, survival depends on the abundance of corals, and butterflyfish populations have declined as a result of bleaching‐induced coral mortality (Wilson et al., 2013). Butterflyfishes alter their diets after coral mortality, decreasing the proportion of bites taken from bleaching‐sensitive *Acropora corals* (Keith et al., 2018; Pratchett et al., 2004; Zambre & Arthur, 2018). However, it is unclear whether changes in butterflyfish diets result from weakening prey preferences, or simply reflect changes in the relative availability or abundance of coral species. This study seeks to determine whether changes in preference strength play a significant part in bottom‐up dietary changes, by directly quantifying changes in the strength of food preferences across a scale of food availability. Here, we use pre‐ and post‐bleaching data on foraging behaviour to determine how dietary breadth, preference strength and niche partitioning are affected following extensive coral mortality. We quantify the extent to which foraging behaviour of coral‐feeding fishes changes in response to decreases in food availability following coral bleaching. Specifically, we test the hypotheses that (*1*) when food availability decreases, dietary preferences of fish decline and that (*2*) weakened dietary preferences lead to increased dietary overlap (i.e. decreased niche partitioning) among competing species (Table S1).

## 2 MATERIALS AND METHODS

### 2.1 Study system

Our study was conducted on the reef flats and crests (1–3 m depth) at three sites on the north/northwest coastline of Iriomote, Japan; Nata (24.43 N, 123.79 E), Sonai (24.38 N, 123.75 E), and Unarizaki (24.43 N, 123.76 E) (Figure S1). Data were collected for 3 years: 27 May to 11 June 2016, 13 July to 17 July 2017 and 12 July to 18 July 2018. In May and June 2016, there was a mild, but notable thermal anomaly around Iriomote, elevating sea surface temperatures 1.0–1.5°C above the 30‐year mean. Combined with unusually calm conditions, this resulted in widespread coral bleaching, with the first signs noticeable to observers on the final day (June 11) of sampling in 2016. At the time of completion of the 2016 surveys there had been no coral mortality; however, 1 year later, we observed coral mortality of ~65% (Baird et al., 2018; Keith et al., 2018). These temperature differences alone are unlikely to have a substantial effect on behaviour; behavioural changes of adult reef fishes have only been documented at temperature increases of 3°C or higher above ambient conditions (ex: Allan et al., 2015). As our focus is specifically on the effect of coral mortality, rather than bleaching itself, we are confident in establishing the 2016 surveys as ‘pre‐mortality’.

### 2.2 Sampling butterflyfish and coral assemblages

We estimated butterflyfish and coral abundances using six 50‐m transects at each site, per year. The same 50‐m transect placements were used for both the butterflyfish and coral counts, and the butterflyfish counts were completed first to limit any effect of diver presence. As transect positioning was constrained by the size and shape of the site, they were consistent across the years. Transects were placed at approximately 1 m depth parallel to the reef crest. This placement focused on the areas of the reef most heavily used by coral‐feeding butterflyfishes, and it was in the same area where our feeding observations were conducted. To estimate butterflyfish abundance, we counted and recorded the identity of every butterflyfish within 2.5 m on either side of the transect (50 m × 5 m belt transects, English et al., 1997). To estimate coral cover, we recorded the benthic substrate every 0.5 m along the transect, identifying hard (scleractinian) corals to species (50‐m point intercept transects, Rogers et al., 1983). We recorded a total of 107 hard coral species on benthic transects; 60 in 2016, 48 in 2017 and 55 in 2018. There was a significant difference in the number of coral species per transect among years (ANOVA: *df* = 2, *F* = 7.604, *p* = 0.001), with the number of species decreasing in 2017, then increasing slightly in 2018. In 2016 there were 13.7 ± 3.8 coral species per transect, 9.1 ± 2.9 per transect in 2017 and 11.0 ± 3.3 per transect in 2018. There was a significant difference in total butterflyfish abundance among the 3 years, with abundance decreasing in 2018 (Table S3, Kruskal–Wallis: *df* = 2, 𝜒^2^ = 17.05, *p* < 0.001). Total butterflyfish abundance was 175 in 2016 (mean = 11.7 ± 5.2 per transect), 223 in 2017 (12.4 ± 5.7 per transect) and 102 in 2018 (5.7 ± 3.4 per transect). Changes in total butterflyfish abundance occurred without substantial change in the rank‐order of fish species, with the most abundant species, *Chaetodon lunulatus*, consistent across all 3 years of the study. Rank‐orders were strongly correlated between 2016 and 2017 (*ρ* = 0.80, *p* < 0.001), and between 2017 and 2018 (*ρ* = 0.76, *p* = 0.002).

### 2.3 Sampling butterflyfish diets

To estimate butterflyfish diets, we followed a focal fish on either snorkel or SCUBA for 3 min and recorded every bite they took on the substrate (following Berumen et al., 2005) along with the species of scleractinian corals that were bitten (following Pratchett, 2005). Over the 3 years, we recorded the diets of 485 individual fishes (approx. 24 hr of direct observation) from five different species based on 11,640 bites on hard coral tissue (Table S4). Previous work has shown that three‐minute observations are sufficient to sample butterflyfish diets and that longer observations do not record significantly more prey species (Berumen, 2001). Butterflyfish are appropriate for on‐site behavioural analysis as they are minimally disturbed by diver presence (Kulbicki, 1998). During the observation period, the observer maintained a distance greater than the fish's perceived flight initiation distance (Ydenberg & Dill, 1986), generally 2–4 m, and the observer minimized their movement. Many butterflyfish species are pair‐forming, so to avoid dependence in our observations, only one of the two fish from each pair was observed. To avoid repeat sampling of the same fish, the observer travelled along the reef in a *U*‐shaped search pattern (Chidlow et al., 2006), which prevents observers from moving back through areas already sampled. The width of the *U*‐shape was approximately twice the width of the average foraging territory (varied between species) (ex: 60–170 m^2^ [18–30 m width] for *C. lunulatus*, Berumen & Pratchett, 2006). Moreover, the observer sought to complete all observations for a given species on 1 day, to avoid repeat sampling of the same individual on separate days. All feeding observations were performed by a single observer (AH Baird), and the following contextual variables were recorded for each observation: date, time, and weather. All sampling occurred between 08:00 and 16:00, with daily observation times limited by tide height (>1 m). In over 800 hr in the field, we did not observe a single predation event on butterflyfishes, consistent with the known scarcity of these occurrences (Ehrlich, 1975). As such, predation did not pose a major factor in butterflyfish behaviour during these observations. All data were collected through direct, in situ observation and behavioural observations were performed in a way to ensure minimal fish stress. This work was approved by Lancaster University's Animal Welfare and Ethical Review Body (AWERB).

### 2.4 Data analysis and hypothesis testing

We evaluate two primary hypotheses to determine whether coral‐feeding fishes experience bottom‐up changes in foraging behaviour after bleaching and evaluate the effect these changes have on the corallivore community. (Hypothesis 1) Butterflyfish should exhibit weakened dietary preferences in response to decreasing food availability. To test this, we used two complementary analyses (Table S1). First, we compared changes in the evenness of observed diets against changes the evenness of coral assemblages (1A). If changes in dietary evenness are not accompanied by significant changes in coral evenness, then changing dietary preferences may also play a role in observed dietary changes. Second, to test for a change in the strength of dietary preferences (1B), we quantified whether losses in coral cover affected the consistency of dietary preferences among individuals, within species. (Hypothesis 2) Weakened dietary preferences should lead to increased dietary overlap (i.e. decreased niche partitioning) among competing species. To test whether resource overlap increased after coral mortality, we calculated resource overlap for each year and used null models to compare the level of resource overlap to random chance.

### 2.5 Evenness of fish diets given altered coral assemblages

To test hypothesis 1A, we evaluated whether diets increased in evenness, exceeding an expected level of change based on changes to coral assemblages. The observed dietary breadth of a species may be more or less evenly distributed as a result of the availability of food items and the strength of its dietary preferences. If the evenness of fish diets increased but was matched with an equivalent increase in the evenness of coral assemblages, then changes in relative resource abundance, rather than weakening dietary preference, would be the likely driver of observed dietary expansion. In contrast, a large increase in the evenness of fish diets, with little change in the evenness of coral assemblages could be attributed to a weakening of dietary preferences.

We compared the evenness of coral assemblages and fish diets over time (i.e. in years before and after disturbance), using Hurlbert's Probability of Interspecific Encounter (PIE) (Gotelli, 2008). When randomly selecting an individual from a community, PIE represents the probability of selecting the same species twice in a row; thereby, it is an intuitive measure of community evenness. All hard coral species were included in the species pool for PIE analyses. For the coral assemblage, PIE was calculated for each transect individually, to use each transect as an independent replicate of coral assemblage structure. For fish diets, we summed the individual observations of a particular fish species from each of the three sites, as each individual observation contained too few prey species to calculate reliable PIE values. From these summed diets, we calculated an overall dietary evenness value for each species at each site and year. This analysis included the four most commonly observed fish species, (*n* = 4 species, Table 1, *Chaetodon citrinellus, C. lunulatus, Chaetodon plebeius* and *Chaetodon trifascialis*), which comprise ~80% of all foraging observations. All other species were excluded from the analysis due to limited observations. As PIE values were not normally distributed across years, for both coral assemblages and butterflyfish diets, we tested whether PIE values varied across years with a Kruskal–Wallis test followed by a Dunn's post‐hoc test to determine significant differences among years.

**TABLE 1:** List of all species included in the study. Use of species in certain portions of the analysis was limited by the amount of replicate observations. Four species, listed in grey, were numerically abundant over multiple locations and time‐periods, allowing their inclusion in all steps of the analysis. Coral‐feeding designations taken from Cole & Pratchett, 2014. Percentages of *Acropora* in diets calculated based on the number of bites, summed across all individuals of a species in each year

Species	Obligate versus facultative	Proportion of *Acropora* in diet 2016	Proportion of *Acropora* in diet 2017	Proportion of *Acropora* in diet 2018	Included In…	
					Diet plasticity tests	Resource overlap models
*Chaetodon citrinellus*	Facultative	39% 699 of 1,770 bites	9% 66 of 771 bites	11% 77 of 723 bites	✓	✓
*Chaetodon lunulatus*	Obligate	51% 1033 of 2,034 bites	14% 131 of 931 bites	17% 252 of 1,489 bites	✓	✓
*Chaetodon plebeius*	Obligate	12% 34 of 290 bites	0% 0 of 190 bites	0% 0 of 145 bites	✓	✓
*Chaetodon rafflesii*	Facultative	3% 4 of 145 bites	1% 4 of 382 bites	0% 0 of 129 bites		✓
*Chaetodon trifascialis*	Obligate	94% 2273 of 2,432 bites	50% 445 of 885 bites	61% 364 of 599 bites	✓	✓

### 2.6 Quantifying changes in intraspecific dietary preferences

To test hypothesis 1B, we evaluated whether losses in coral cover were associated with a weakening of dietary preferences. We did so by calculating whether individuals within a species showed a consistent ranking in selectivity values for different corals, and whether this consistency in preferences varied with total coral cover. In our framework, a species has strong dietary preferences if all individuals of the species consistently preferred the same coral taxa. For any species, the more consistent its ranked order of coral preferences is among individuals, the stronger its dietary preferences are overall. We used dietary data from the individual observations to calculate this ranked order of preferences for the seven most abundant coral genera in the dataset (*Acropora, Favites, Galaxea, Goniastrea, Montipora, Pocillopora* and *Porites*).

For this analysis, preferences at the coral species level would be too finely subdivided to be informative (i.e. rare coral species would show large fluctuations in selectivity with only minor changes in bites), so bites were grouped at the genera level. We calculated the selectivity of each individual fish for each of these seven genera using Ivlev's Electivity Index (Ivlev, 1961). This metric compares the relative consumption (proportion of bites) on each food type to its relative abundance (proportion of coral cover). It indicates the degree to which a forager seeks out particular food items and scales from complete avoidance (−1) to exclusive selection (1) (Ivlev, 1961). An electivity value of 0 would indicate an individual fed randomly on a coral genus, in proportion with its abundance. These seven coral genera include >90% of all bites on hard coral. All other genera were consumed too infrequently to generate reliable selectivity values. This analysis included the same commonly observed fish species, as above (Table 1, *n* = 4 species).

**FIGURE 1:**
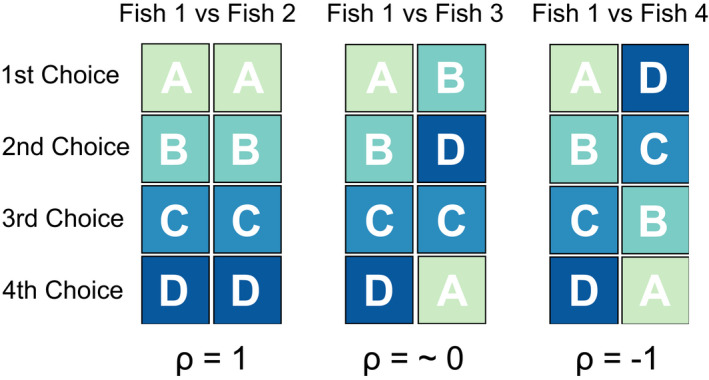
Schematic of dietary preference comparisons. Food items (coral genera, lettered A–D) were ranked from most to least preferred based on Ivlev's Electivity Index. Preference rankings of an individual fish were compared against all others of the same species in the same site and year. They were compared with Spearman's rank correlation, rescaled where *ρ* = 1 reflects the exact same order of preferences, and *ρ* = −1 reflects the exact opposite order of preferences

To determine whether individuals consistently preferred the same corals, we made pairwise comparisons between an individual's dietary selectivity values and those of all other fish of the same species in that same year and site. Pairwise comparisons measured the consistency in rankings of the seven coral genera using Spearman rank‐order correlations. We then rescaled the resulting Spearman correlation coefficients to ensure that all values were positive and matched the beta distribution, which is well suited to response variables bounded in both directions (Ospina & Ferrari, 2010). The rescaling performed was as follows: (*y*_1_ = [*y*_0_ + 1]/2), such that our coefficients scaled from 0 to 1. The coefficient for two individuals with the opposite order of food preferences is 0; whereas the coefficient for two individuals with the same order of preferences is 1 (Figure 1). These values are hereafter referred to as ‘Preference Coefficients’ and reflect the strength of dietary preferences. In this way, a mean Preference Coefficient (i.e. average of all Spearman correlation coefficients) close to 1 indicates strong adherence to a consistent ranking of dietary preferences, and a mean coefficient close to 0.5 indicates no consistent ranking. These values only reflect dietary selection on hard corals, not other prey items such as non‐coral invertebrates, to focus on changes in hard coral consumption after the coral mortality event.

We used a generalized linear mixed model with a beta distribution (Beta GLMM) to model the strength of dietary preferences as a function of coral cover. Specifically, we used a one‐inflated beta distribution for the model. This model structure is well suited to fit data when the response value is bounded, 0 < *Y* ≤ 1 (Ospina & Ferrari, 2010). Our model assesses the strength of dietary preferences (i.e. Preference Coefficient) as a function of total hard coral cover at the site level, with a fixed covariate for forager species. Each observation is a pairwise comparison between two individual fish, so there was dependency among observations that shared a common fish individual. To account for this dependency, we used the identity of each of the two fish in the comparison as two separate random intercept effects. The optimal model was determined by backward model selection using Aikaike information criterion (AIC), sequentially dropping terms from an initial full model (following Zuur et al., 2007), which also included fixed covariates of site and year. At each stage of model selection, two models were compared, and the more complex model was only preferred if it improved AIC by two or more. All models were run in R 3.6.1 (R Core Team, 2019) using the package gamlss (Rigby & Stasinopoulos, 2005), which supports the one‐inflated beta distribution. While package gamlss typically generates additive models, it will generate a linear model if no smoother is set. Details on model selection are listed in Table S2. We predicted the preference coefficient for each species within the range of coral cover values for which it was observed. We generated 95% confidence intervals around these predictions using jackknife resampling, whereby we randomly subset the dataset to 80% of the observations, re‐fitted the model and generated predictions on this subset, and repeated the process for 100 replicates (McIntosh, 2016). These 100 jackknife predictions were ordered, and confidence intervals taken as the 5th and 95th values. Predictions were made specifically on one level of the random intercept effect. To test whether the rate of change in preference strength varied among species, we fit an alternate model with an interaction between hard coral cover and butterflyfish species. We then compared the two models via AIC, as above. Lastly, to determine whether changes in preference strength were solely driven by changes in rankings relative to *Acropora*, we re‐ran the optimal model on a subset of the selectivity data, excluding *Acropora*, to see if effects of food availability remained significant.

### 2.7 Influence of disturbance on niche partitioning among coral‐feeding fishes

To test the effect of disturbance on resource partitioning, we measured the Pianka niche overlap index (Gotelli & Ellison, 2013) of resource‐use before coral mortality (2016) and at 1 year (2017) and 2 years after coral mortality (2018). For any two fish species, the Pianka niche overlap index represents the proportion of resources used by either species that are shared by the two; values range from 0 (no shared resources) to 1 (all resources shared; Pianka, 1973). For each year we generated a resource‐use matrix lists butterflyfish species (rows) against the top seven coral genera (columns). Matrices included the same seven coral genera and four fish species as above, with the addition of *C. rafflesi* (Table 1, *n* = 5 species), comprising >85% of all foraging observations Values in these matrices are the sum of all bites across an individual butterflyfish species. For a full resource‐use matrix, Pianka niche overlap is the mean pairwise niche overlap between all fish species. Although the proportional comparisons involved in this method do not require equivalent sampling, the fewer observations there are for any species, the greater likelihood of error around the proportional allocations of bites. For this reason, we only included *Chaetodon* species that were observed at least eight times (≥24 min of feeding) in all three time periods. These comprise Pianka values were calculated in EcoSimR (Gotelli & Ellison, 2013).

To account for the fact that Pianka values can vary depending on matrix structure, rather than directly comparing Pianka values between years, the common approach is to compare the value for each year against a null expectation for the given matrix (following Gotelli & Ellison, 2013). For each year, we generated a set of 1000 simulated matrices for comparison with the observed matrix to determine the likelihood of an equivalent degree of resource partitioning observed by chance. Simulated matrices were generated in EcoSimR with the preferred ‘RA3’ algorithm, which maintains the dietary breadth (number of resources) consumed by each fish species but randomly reshuffles the specific resources consumed. Column totals (total bites on each coral species) are allowed to fluctuate. For each year EcoSimR also generates the Standardized Effect Size (SES) of the observed level of partitioning against the null expectation (Gotelli & McCabe, 2002) and corresponding one‐tailed and two‐tailed *p*‐values. SES is calculated as the difference between the observed value and the mean simulated value, divided by the standard deviation of simulated values. *p*‐values are calculated based on the proportion of occurrences that the observed value is less or greater than the simulated value. We computed resource‐use matrices at the genus level for the food resource (i.e. corals) and included all forager species that were observed frequently before and after disturbance.

## 3 RESULTS

### 3.1 Evenness of fish diets given altered coral assemblages

**FIGURE 2:**
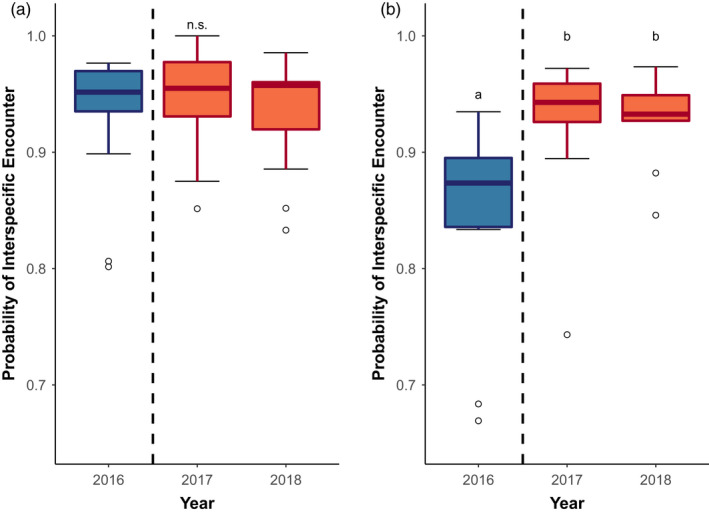
Evenness of corals and diets before and after disturbance. Hurlbert's Probability of Interspecific Encounter for (a) the coral community and (b) butterflyfish diets, before and after bleaching. Dashed lines indicate the coral mortality event. Blue boxes represents these communities before bleaching (2016), orange boxes are at 1 year (2017) and 2 years (2018) after bleaching. Lettering above the box plots indicates significant differences among years

Changes in dietary evenness were not accompanied with significant changes in coral evenness (Hypothesis 1A) and instead were likely driven by weakening of butterflyfish dietary preferences (1B). Fish diets became more even after bleaching (Figure 2b, Kruskal–Wallis: *df* = 2, 𝜒^2^ = 11.30, *p* = 0.004), despite no significant change in the evenness of coral assemblages (Figure 2a, Kruskal–Wallis: *df* = 2, 𝜒^2^ = 0.35, *p* = 0.836). On average, fish diets increased in evenness by 10%. The PIE for fish diets differed between 2016 and both post‐disturbance years (2017 – Dunn: *z* = −3.14, *p* < 0.001; 2018 – Dunn: *z* = −2.63, *p* = 0.004), but PIE did not differ between 2017 and 2018 (Dunn: *z* = 0.452, *p* = 0.326).

### 3.2 Quantifying changes in intraspecific dietary preferences

**FIGURE 3:**
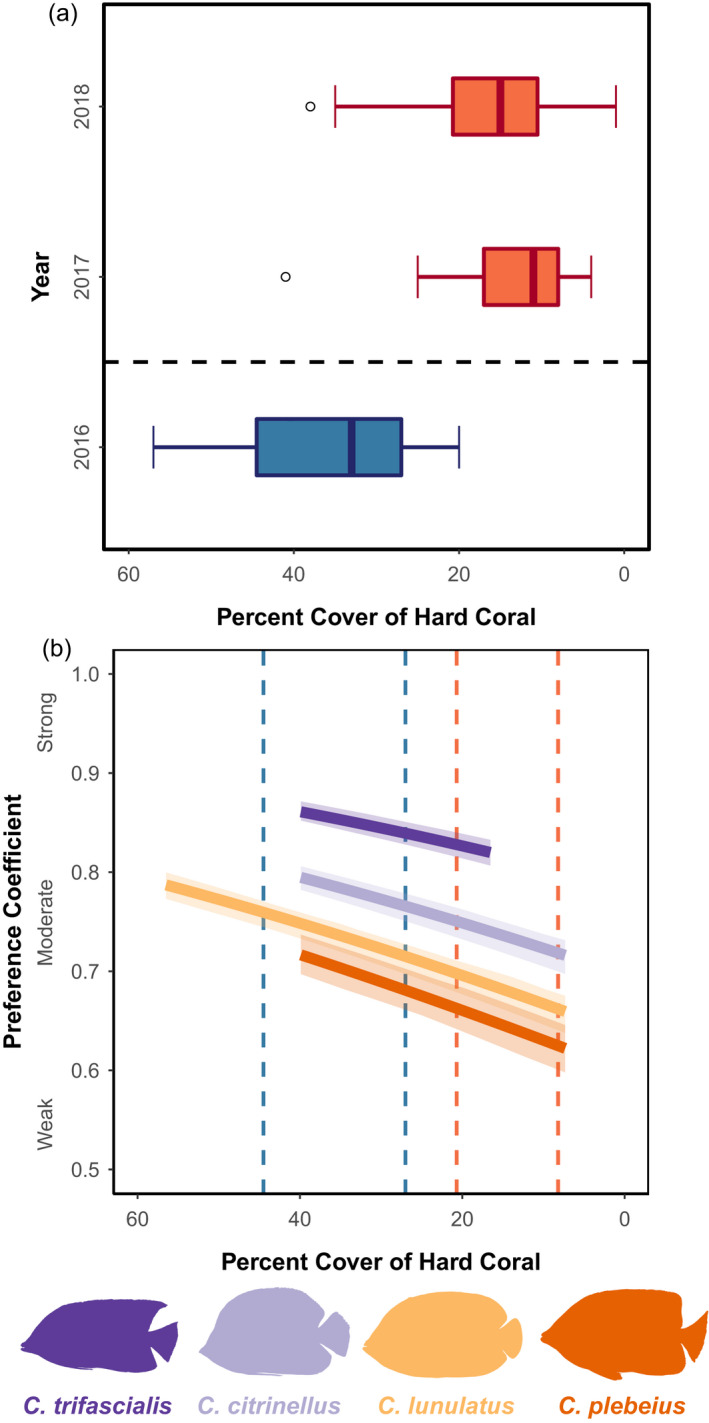
(a) Loss of hard coral cover (%) due to coral bleaching. Transects were set on shallow (1 m) reef crests. (b) Reaction norm plot of dietary plasticity with disturbance, under the linear model. Solid lines are GLMM predictions of Preference Coefficient for each species across the range of observed coral cover, with 95% confidence intervals generated from jackknife resampling of 80% of the entire dataset. Large values for Preference Coefficient reflect a consistent ranked order of food items among individuals of a species. Preference Coefficients plotted here are predicted under just one level of the random effects (one individual fish pair), please refer to the Results for average parameter estimates across all random effect levels. Dashed lines indicate the interquartile range of hard coral cover in the pre‐ (blue) or post‐coral‐mortality (orange) condition, matching panel (a)

When food availability decreased, the dietary preferences of coral feeding fish decreased (Hypothesis 1B). Coral cover decreased sharply by 65% after bleaching in 2016 (Figure 3a), and as coral cover decreased, so did the strength of fishes' dietary preferences (Figure 3b, GLMM coral covariate = 0.79, 95% CI: 0.75–0.82, model *R* ^2^ = 0.51). As a result, coral‐feeding fishes consumed broader diets (i.e. took bites from a broader range of coral species). Fish species also differed significantly in their overall strength of dietary preferences. Regardless of food availability, *C. plebeius* had the weakest preferences (Preference Coefficient estimate: 0.49, 95% CI: 0.47–0.52), with the other fish species exhibiting sequentially stronger coral preferences (*C. lunulatus*: 0.53, 0.52–0.55, *C. citrinellus*: 0.60, 0.58–0.61, *C. trifascialis*: 0.70, 0.68–0.72). These values specifically reflect the strength of preferences among coral prey. The relationship between coral cover and preference strength did not vary among species. An interaction between coral cover and species was not preferred via AIC (∆AIC = 2.78). Lastly, effects of hard coral cover on fish diet preferences were not driven solely by changes in ranking relative to *Acropora*. The effect of hard coral cover remained significant even when *Acropora* corals were excluded (Table S5, coral covariate = 0.58, 95% CI: 0.51–0.64, model *R* ^2^ = 0.38).

### 3.3 Influence of disturbance on niche partitioning among coral‐feeding fishes

**FIGURE 4:**
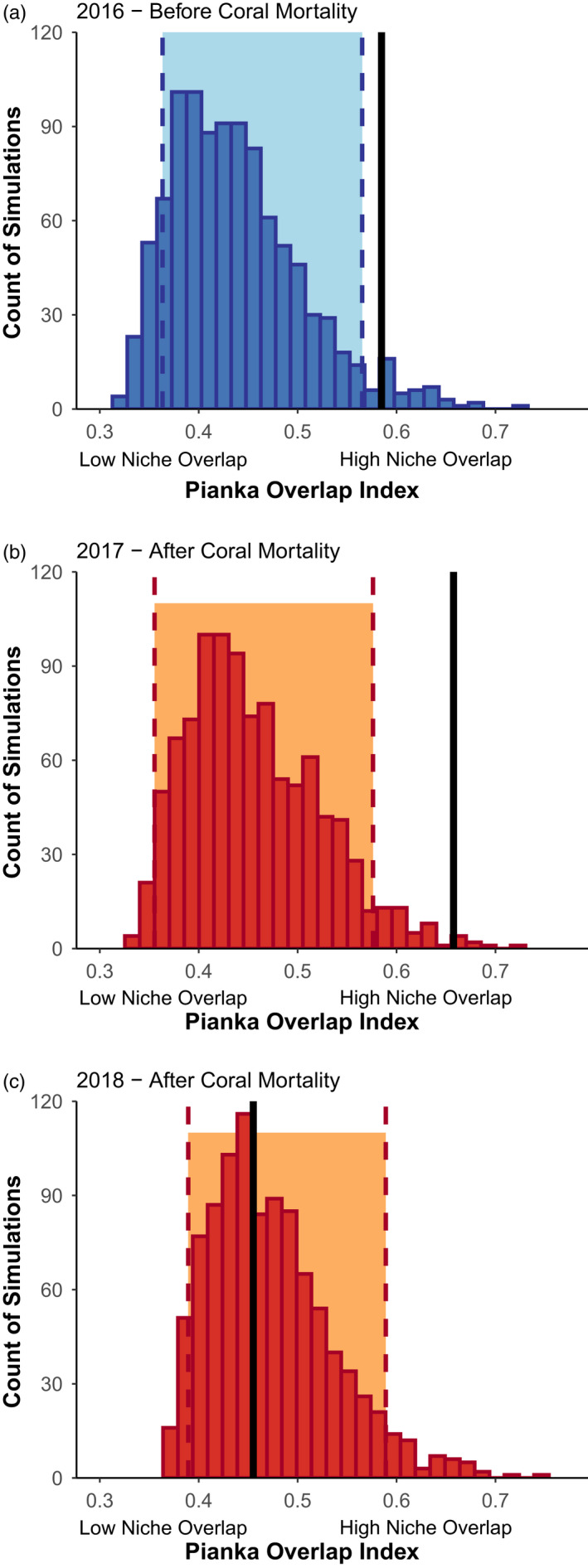
Null model analysis of resource partitioning with EcoSim (A) before coral mortality (blue, 2016) and (B and C) after coral mortality (orange, 2017, 2018). The Pianka overlap index scales from 0 (no shared resources among species) to 1 (all resources shared among species), and an increase represent an increase in average niche overlap. Null matrices were generated with algorithm ‘RA3’. Black vertical lines represent the Pianka overlap index measured before and after disturbance, histograms represent the null expectation for each disturbance

Weakened preferences did not lead to increased dietary overlap among genera of butterflyfishes (Hypothesis *2*). Dietary expansion among foragers was generally associated with a decrease in resource overlap, but with a delayed effect (Table S6). Before bleaching, many species overlapped in their consumption of *Acropora* species, and resource‐use overlapped significantly more than random (Figure 4a, SES = 2.20, *p* [one‐tailed] = 0.041). One year after bleaching, these fish ate less *Acropora*, but simultaneously consumed more *Montipora* species. As a result, in 2017, resource‐use continued to overlap significantly more than random (Figure 4b, SES = 3.00, *p* [one‐tailed] = 0.008). However, 2 years after bleaching in 2018, species consumed a variety of different coral genera, with less overlap on any particular genera. As a result, resource‐use no longer differed from a null expectation of random resource use (Figure 4c, SES = −0.29, *p* [one‐tailed] = 0.547).

## 4 DISCUSSION

Using foraging behaviour data from both before and after a coral bleaching event, we found that coral mortality reduced food availability for butterflyfishes, and that this disturbance caused changes in their behaviour, altering resource use among species. As predicted by theory (Emlen, 1966), after coral cover decreased by 65%, butterflyfishes exhibited increased dietary evenness and weakened prey preferences. All four species showed changes in dietary breadth and, though the strength of their preferences differed initially, the preferences of these species weakened by a similar degree. For example, prior to bleaching, *C. trifascialis* took 94% of its bites from *Acropora* corals, with 37% of its bites from its preferred food source, *Acropora hyacinthus*. Prior to bleaching, it only took 3% of its bites from *Montipora spp*. However, after bleaching, *Acropora* corals only comprised 55% of its diet, with *A. hyacinthus* only comprising only 16% of bites. After bleaching, *C. trifascialis* instead consumed a number of alternative *Acropora* (38%) and *Montipora* (39%) species. Furthermore, while there were changes in relative prey abundance (Table S7), increasingly even fish diets reflected a degree of change beyond the nonsignificant increase in evenness of the coral community. Instead, this weakening of prey preferences closely follows predictions from optimal foraging theory, where a forager's niche breadth expands in response to reduced food availability (Emlen, 1966). A less consistent ranked order of preferences is also reflective of the niche variation hypothesis, wherein expanded diets at the species level are brought about by greater variation in diets among individuals (Bolnick et al., 2010).

Contrary to theoretical expectations (Del Moral, 1985; Økland, 1990; Watson, 1980), weakened dietary preferences resulted in less resource overlap at the genera level. This may be due to the unique pre‐bleaching structure of the coral‐corallivore network. Coral assemblages are often dominated by bleaching‐sensitive *Acropora* species (Renema et al., 2016), which provide the majority of coral tissue consumed by butterflyfishes (Keith et al., 2018). As they are more susceptible to bleaching, *Acropora* corals often face the greatest degree of mortality (Loya et al., 2001; Marshall & Baird, 2000). Following coral mortality, nearly all butterflyfish species decreased their consumption of bleaching‐sensitive *Acropora* corals and increased their consumption of a number of other genera. This led to a reduction in resource overlap among species at the genera level. Thus, while dietary preferences became weaker, species partitioned the remaining resources to a greater degree than before mortality, coinciding with previously documented reductions in inter‐specific aggression (Keith et al., 2018).

Our results show butterflyfishes are quite flexible in their diet selection in the short term, but recovery trajectories indicate bleaching‐induced coral mortality has a profound and long‐lasting effect on food availability for corallivorous fishes. Recovery time estimates for a bleached reef range from 7–29 years without disturbance (Gouezo et al., 2019; Robinson et al., 2019). However, bleaching events have become a frequent occurrence, with the average bleaching recovery window shortening from 27 years in the early 1980s to 6 years in 2016 (Hughes et al., 2018). Although lifespan data are limited, butterflyfish often live over 10 years, so it is unclear whether the behavioural changes seen will persist beyond the current generation, and how this could affect butterflyfish populations (Berumen et al., 2005, Nowicki et al., 2018). Butterflyfish population sizes significantly decline following coral mortality events, particularly obligate coral feeders (Wilson et al., 2013); however, the resource flexibility adopted by these fishes may be an important factor in their long‐term persistence. In addition, species extirpations and population losses may be a factor in how long these dietary changes continue, as increases in dietary breadth can also stem from decreased competitor abundance (‘ecological release’, Bolnick et al., 2010). Understanding dietary changes among butterflyfish species is an important step in determining their relative competitive ability, which will help predict species persistence under continued change.

Behavioural responses to anthropogenic disturbances can have wide‐reaching impacts on resource competition within communities. Coral mortality following bleaching is one of many human‐induced disturbances that can bring about drastic shifts in foraging behaviour (Keith et al., 2018; Samways, 2005; Thompson et al., 2019). For corallivores, coral mortality caused a major decrease in food supply and weakened dietary preferences. This simultaneous dietary expansion of multiple species occurred in such a way that all species reduced their consumption of bleaching‐sensitive *Acropora*, and instead consumed a wide variety of other coral genera, resulting in a decreased resource overlap. While behavioural changes may increase resilience in the short term, coral mortality has reduced populations of coral‐feeders world‐wide, so these changes may not be sufficient to ensure long‐term resilience of butterflyfishes on coral reefs. To better understand and manage the effects of disturbance events, we must go beyond species composition to consider their effects on foraging behaviour, which may alter competition and long‐term community stability.

## AUTHOR CONTRIBUTIONS

Robert F. Semmler, Andrew H. Baird, and Sally A. Keith proposed dietary analysis methods. Nathan J. Sanders and Paul J. CaraDonna proposed community analysis methods and supervised their usage. Robert F. Semmler performed all data analyses and wrote the first draft of the manuscript, all authors contributed substantially to revisions. Andrew H. Baird collected all foraging observation/coral cover data. James P. W. Robinson and Xin Jing assisted with model fitting and community analyses. All authors gave final approval for publication.

## CONFLICT OF INTEREST

Co‐authors Nathan J. Sanders and Paul J. CaraDonna are Editors at Journal of Animal Ecology, but took no part in the peer review and decision‐making processes for this paper.

## Supporting information


SupplementS1

## Data Availability

Data are available on FigShare https://doi.org/10.6084/m9.figshare.20415849 (Semmler, 2022).
