# Supplementary material for: Reef fishes weaken dietary preferences after coral mortality, altering resource overlap
Source: J Anim Ecol. 2022 Aug 16;91(10):2125–34. doi: 10.1111/1365-2656.13796 (PMC9804366; doi:10.1111/1365-2656.13796)
Supplement: Supplementary file 1 — SupplementS1 [file JANE-91-2125-s001.docx]

**Supplemental Materials**


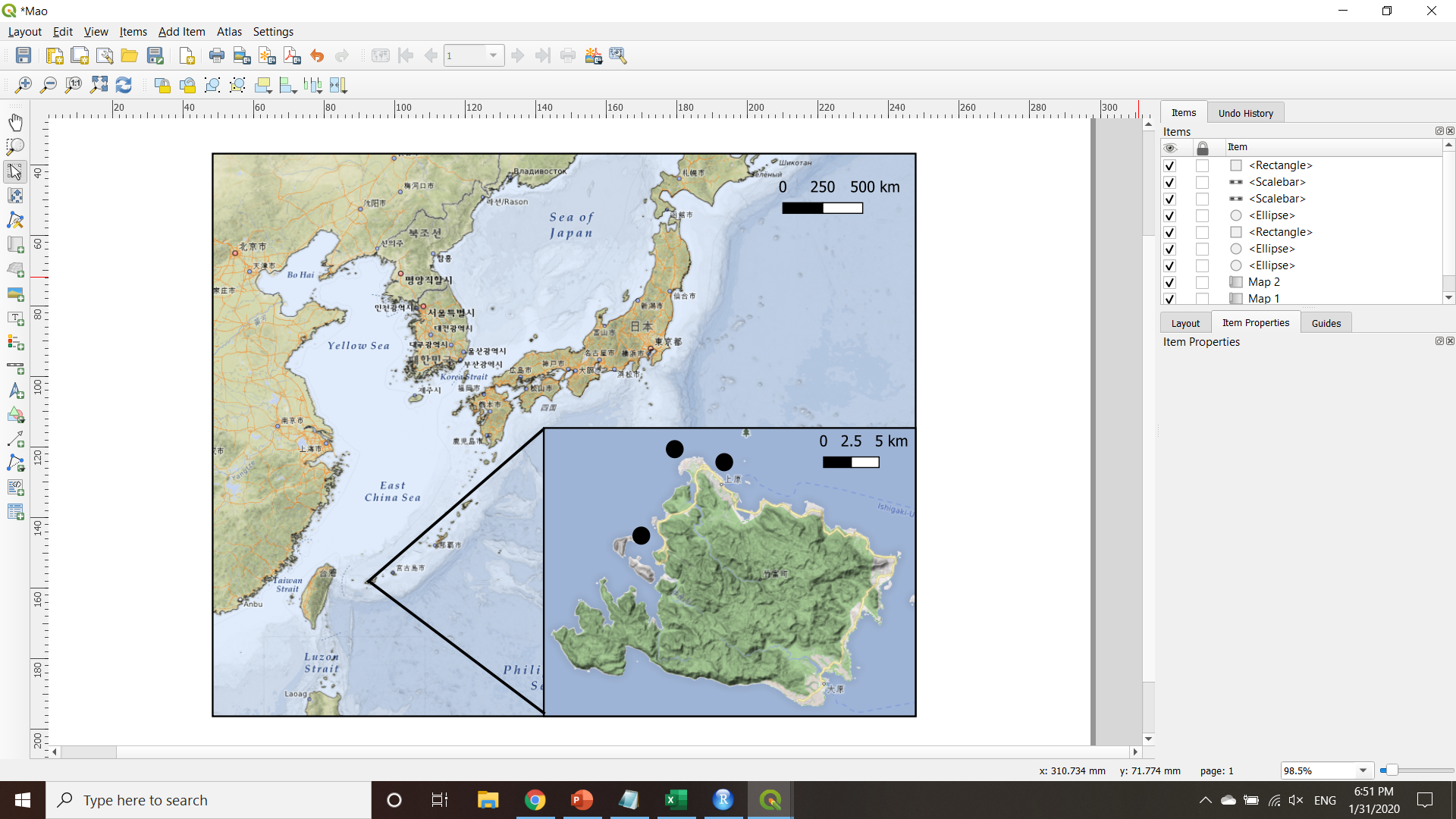


Figure S1: Map of Iriomote, Japan. Main panel shows Iriomote’s location in the East China Sea, near Taiwan. Black circles on the inset indicate the three reef locations (from West to East): Sonai, Unarizaki, and Nata.


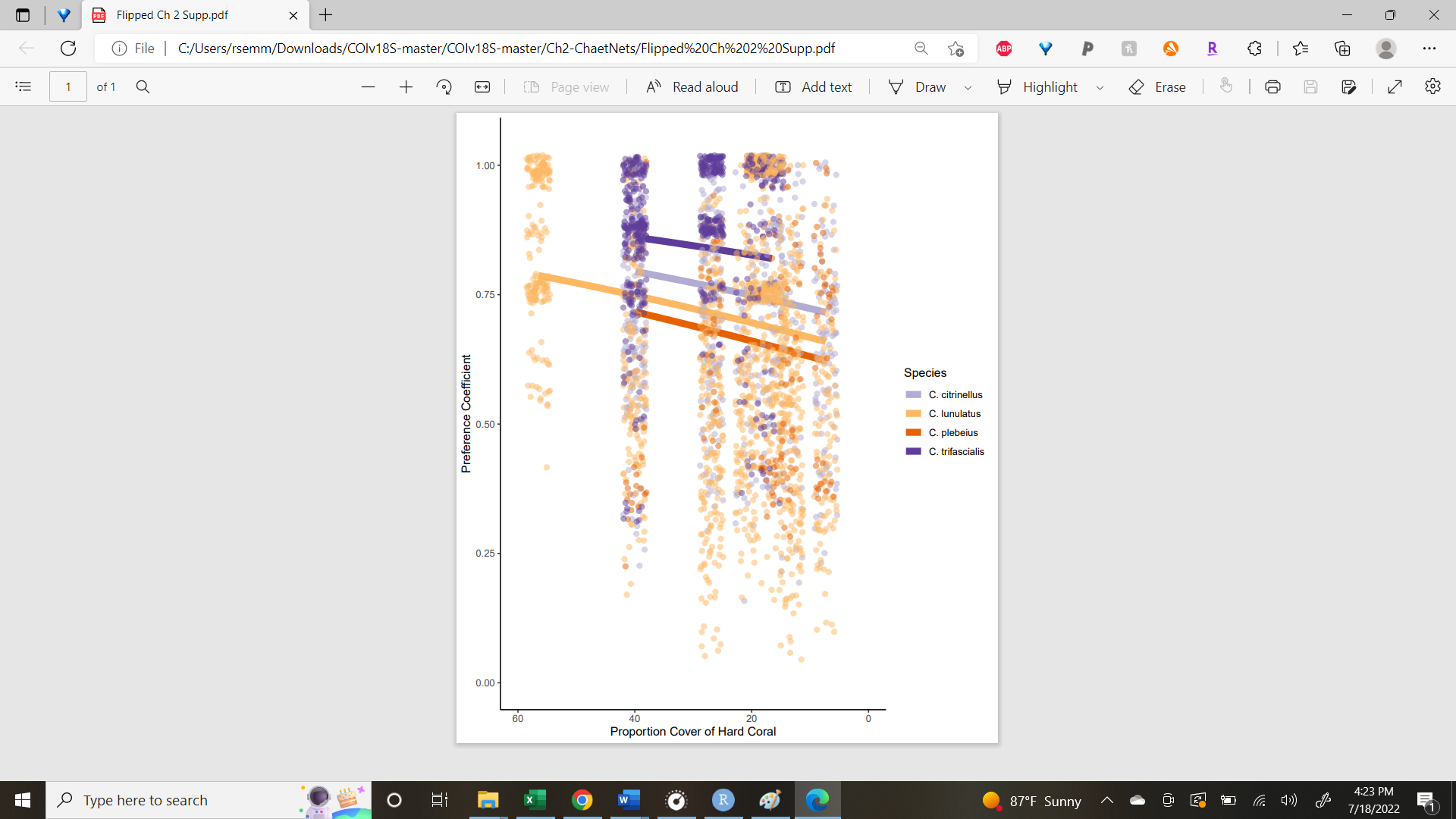


Figure S2: Predicted Preference Coefficients against raw data for figure 2B (n = 2,964 points). As there are many comparisons with the same Preference Coefficient or coral cover, these are jittered to show the relative density of observations.


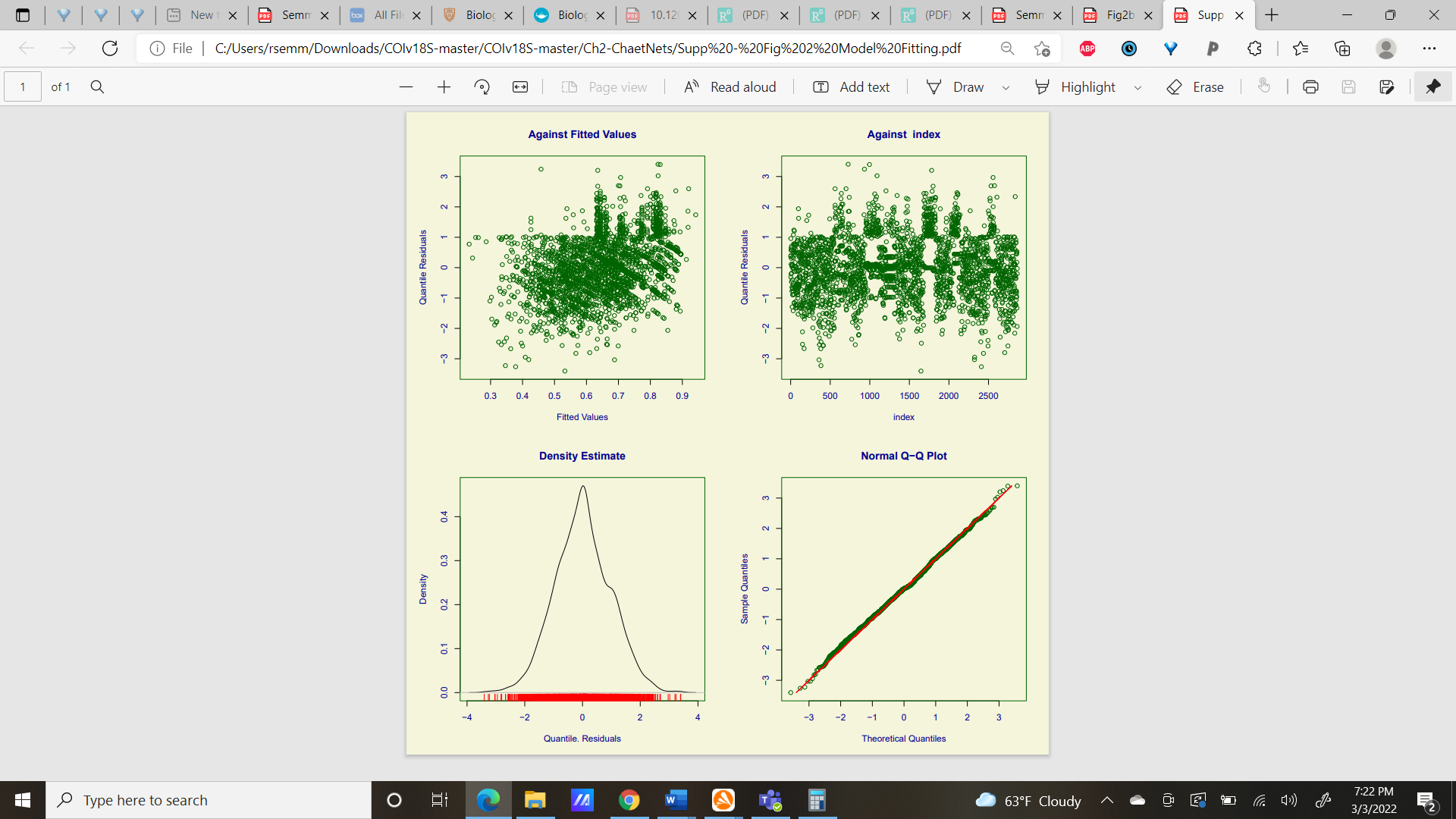


Fig S3) Preference Coefficient Model Fit, gaps and clumps in residuals in the top plots are a result of some preference coefficients not being theoretically possible with the nature of the data. Ranked preferences for 7 coral genera can only be ordered a limited number of ways, so there are a limited number of Preference Coefficients possible when comparing any two ranked orders.

Table S1) Primary hypotheses tested in this analysis, the tests and methods used to address these, and the figures that illustrate each result.

| **Hypothesis** | **Subsection** | **Figure** | **Test** | **Result** |
| --- | --- | --- | --- | --- |
| 1) Coral feeding fish will expand their diets after coral mortality, consuming wider diets as a result of weakening dietary preferences | 1A) Changes in dietary evenness after bleaching are not accompanied by equal changes in the relative abundance of food items (i.e., coral evenness) | Figure 2 | Evenness (PIE) of diets vs. evenness of coral assemblages (Kruskal-Wallis + Dunn’s Test) | Diets significantly more even after coral mortality, no significant change in coral evenness |
|  | 1B) Dietary preferences will weaken as food availability (coral cover) decreases | Figure 3 | Preference Coefficient -Consistency of Preferences within Species (GLMM) | Preferences weaken (less consistent) as coral cover decreases |
| 2) Expanded diets will lead to greater niche overlap among species | NA | Figure 4 | Pianka Niche Overlap Index – Null Model Comparison (EcoSim) | Two years after bleaching, niche overlap actually decreases at the genera level, with species partitioning the remaining resources |

Table S2) Details of model selection for Preference Coefficients (Figure 2B). All models are GLMMs produced in package ‘gammlss’. All contain two random intercept effects for fish identity of the two fish in each comparison.

| **Step** | **Model Description (Fixed Effects)** | **AIC** |
| --- | --- | --- |
| 1) | Preference Coefficient ~ CoralCover + Species + Site + Year | 156.26 |
| 2) | Preference Coefficient ~ CoralCover + Species + Site | 154.34 |
| 4) | Preference Coefficient ~ CoralCover + Species | 151.45 |

Table S3) Total abundance of butterflyfish species on transects, by year. Note that only 15 butterflyfish transects were run in 2016, whereas 18 were run in 2017 and 2018. Species in bold text were the most commonly sampled in dietary surveys and were included in our analyses.

| **Species** | **2016** | **2017** | **2018** |
| --- | --- | --- | --- |
| C. argentatus | 3 | 16 | 1 |
| C. baronessa | 0 | 2 | 0 |
| C. bennetti | 1 | 2 | 1 |
| **C. citrinellus** | **11** | **29** | **26** |
| C. kleinii | 3 | 2 | 0 |
| **C. lunulatus** | **115** | **110** | **42** |
| C. melannotus | 2 | 2 | 2 |
| C. ornatissimus | 4 | 8 | 3 |
| **C. plebeius** | **8** | **13** | **2** |
| **C. rafflesii** | **11** | **11** | **4** |
| C. reticulatus | 0 | 0 | 0 |
| C. speculum | 0 | 3 | 4 |
| **C. trifascialis** | **10** | **10** | **4** |
| C. unimaculatus | 7 | 15 | 13 |
|  |  |  |  |
| **Top 5 Followed** | **155** | **173** | **78** |
| **All Species** | **175** | **223** | **102** |

Table S4) Total sample size of focal fish follows across years for species, and sites.

| **Species** | **2016** | **2017** | **2018** |
| --- | --- | --- | --- |
| ***C. citrinellus*** | **51** | **37** | **29** |
| ***C. lunulatus*** | **60** | **52** | **60** |
| ***C. plebeius*** | **16** | **21** | **11** |
| ***C. rafflesii*** | **9** | **22** | **8** |
| ***C. trifascialis*** | **64** | **27** | **18** |
|  |  |  |  |
| **Total** | **200** | **159** | **126** |
|  |  |  |  |
| **Site** | **2016** | **2017** | **2018** |
| Nata | 73 | 57 | 53 |
| Sonai | 75 | 48 | 31 |
| Unarizaki | 90 | 81 | 53 |

Table S5) Model Coefficients for the optimal model, first on all seven coral genera, then excluding *Acropora*. Values in parenthesis are 95% CI’s for parameter estimates. Even excluding changes in rankings relative to *Acropora* (understandably an important driver of this effect), there was still a significant relationship between hard coral cover and preference coefficient.

| Factor | **All Genera R^2^ = 0.51** | **Excluding Acropora**  **R^2^ = 0.38** |
| --- | --- | --- |
| Coral Cover | 0.79 (0.75-0.82) p < 0.001 | 0.58 (0.51-0.64) p = 0.017 |
| *C. citrinellus* | 0.60 (0.58-0.61) p < 0.001 | 0.61 (0.59-0.63) p < 0.001 |
| *C. lunulatus* | 0.53 (0.52-0.55)  p < 0.001 | 0.55 (0.54-0.57) p < 0.001 |
| *C. plebeius* | 0.49 (0.47-0.52)  p = 0.575 | 0.52 (0.49-0.55) p = 0.135 |
| *C. trifascialis* | 0.70 (0.68-0.72)  p < 0.001 | 0.62 (0.58-0.61) p < 0.001 |

Table S6) Proportion of bites on top seven coral genera by year and butterflyfish species. Cells colored by proportion from low (red) to high (green).

| Year | Species | Acropora | Favites | Galaxea | Goniastrea | Montipora | Pocillopora | Porites |
| --- | --- | --- | --- | --- | --- | --- | --- | --- |
| 2016 | citrinellus | 0.59 | 0.02 | 0.00 | 0.08 | 0.12 | 0.16 | 0.03 |
| 2016 | lunulatus | 0.54 | 0.00 | 0.06 | 0.04 | 0.17 | 0.06 | 0.12 |
| 2016 | plebeius | 0.19 | 0.34 | 0.15 | 0.15 | 0.04 | 0.09 | 0.04 |
| 2016 | rafflesii | 0.06 | 0.07 | 0.25 | 0.18 | 0.13 | 0.27 | 0.04 |
| 2016 | trifascialis | 0.94 | 0.00 | 0.00 | 0.00 | 0.03 | 0.03 | 0.00 |
|  |  |  |  |  |  |  |  |  |
| 2017 | citrinellus | 0.16 | 0.03 | 0.00 | 0.09 | 0.58 | 0.14 | 0.01 |
| 2017 | lunulatus | 0.17 | 0.03 | 0.08 | 0.09 | 0.45 | 0.09 | 0.10 |
| 2017 | plebeius | 0.00 | 0.16 | 0.27 | 0.11 | 0.27 | 0.17 | 0.01 |
| 2017 | rafflesii | 0.02 | 0.01 | 0.38 | 0.01 | 0.20 | 0.37 | 0.00 |
| 2017 | trifascialis | 0.52 | 0.00 | 0.00 | 0.00 | 0.46 | 0.02 | 0.00 |
|  |  |  |  |  |  |  |  |  |
| 2018 | citrinellus | 0.17 | 0.03 | 0.00 | 0.11 | 0.28 | 0.29 | 0.12 |
| 2018 | lunulatus | 0.19 | 0.03 | 0.11 | 0.11 | 0.32 | 0.10 | 0.14 |
| 2018 | plebeius | 0.00 | 0.28 | 0.18 | 0.26 | 0.01 | 0.13 | 0.14 |
| 2018 | rafflesii | 0.00 | 0.11 | 0.02 | 0.00 | 0.00 | 0.86 | 0.00 |
| 2018 | trifascialis | 0.60 | 0.00 | 0.01 | 0.00 | 0.31 | 0.08 | 0.00 |

Table S7) Relative coral cover among the top seven. Cells colored by percentage from low (red) to high (green).

| Year | Acropora | Favites | Galaxea | Goniastrea | Montipora | Pocillopora | Porites |
| --- | --- | --- | --- | --- | --- | --- | --- |
| 2016 | 53.66% | 2.37% | 2.27% | 4.12% | 14.32% | 2.88% | 20.39% |
| 2017 | 19.06% | 1.77% | 3.14% | 9.04% | 40.86% | 6.09% | 20.04% |
| 2018 | 12.91% | 2.32% | 2.65% | 6.62% | 60.93% | 2.65% | 11.92% |
|  |  |  |  |  |  |  |  |
